# Postoperative Septic Shock After Esophagectomy for Esophageal Cancer: Risk Factors and Impact on Short- and Long-Term Survival

**DOI:** 10.3390/jpm16050251

**Published:** 2026-05-04

**Authors:** Patricia Piñeiro, Francisco Sánchez, Alberto Calvo, María Tudela, Silvia Ramos, Sergio García, Pilar Benito, Isabel Solchaga, Raquel Vela, Claudia Menéndez, Eneko Cabezuelo, Ignacio Garutti

**Affiliations:** 1 Department of Anesthesiology and Critical Care, Gregorio Marañón University Hospital, 28007 Madrid, Spain; patricia.pineiro@salud.madrid.org (P.P.); f.sanchez@salud.madrid.org (F.S.); carlosalberto.calvo@salud.madrid.org (A.C.); silvia.ramos@salud.madrid.org (S.R.); sgarciaramos2@salud.madrid.org (S.G.); pbenito@salud.madrid.org (P.B.); isabel.solchaga@salud.madrid.org (I.S.); raquelcarolina.vela@salud.madrid.org (R.V.); claudia.menendez@salud.madrid.org (C.M.); eneko.cabezuelo@salud.madrid.org (E.C.); 2 Gregorio Marañón Health Research Institute (ISGM), 28007 Madrid, Spain; 3 Department of Surgery, Gregorio Marañón University Hospital, 28007 Madrid, Spain; maria.tudela@salud.madrid.org; 4 School of Medicine, Complutense University, 28040 Marid, Spain

**Keywords:** esophagectomy, septic shock, postoperative complications, hypoalbuminemia, survival

## Abstract

**Background**: Esophagectomy is associated with substantial postoperative morbidity, with infectious complications remaining a leading cause of mortality. Septic shock represents the most severe infectious complication; however, data on its perioperative predictors and long-term impact after esophagectomy are limited. **Methods**: We conducted a retrospective observational study including consecutive adult patients who underwent esophagectomy with curative intent for esophageal cancer between January 2015 and December 2024 at a tertiary referral center. Postoperative septic shock was defined according to Sepsis-3 criteria. Demographic, clinical, surgical, laboratory, and oncological variables were analyzed. Independent risk factors for septic shock were identified using multivariate logistic regression. Overall survival was assessed using Kaplan–Meier analysis. **Results**: Among 106 patients, 19 (17.9%) developed postoperative septic shock. These patients had a lower body mass index, reduced preoperative and postoperative albumin levels, and a higher incidence of advanced lymph node involvement. Septic shock was strongly associated with severe postoperative complications, including anastomotic leakage, hemorrhagic shock, acute respiratory distress syndrome, acute kidney failure, and increased rates of PICU readmission. In multivariate analysis, lower albumin levels at PICU admission (OR 0.54; 95% CI 0.29–0.99) and advanced nodal stage (OR 4.98; 95% CI 1.36–18.3) were independently associated with the development of septic shock. Patients who developed septic shock had significantly higher in-hospital mortality (31.6% vs. 1.1%, *p* < 0.001) and markedly reduced long-term survival, even among those discharged alive. **Conclusions**: Postoperative septic shock after esophagectomy is a devastating complication with a profound negative impact on both short- and long-term survival. Hypoalbuminemia and advanced lymph node involvement are independent predictors of septic shock. These findings support the integration of simple clinical and laboratory markers into personalized perioperative risk stratification models, enabling individualized management strategies to reduce severe postoperative complications.

## 1. Introduction

Esophageal cancer is the eighth most commonly diagnosed malignancy and the sixth leading cause of cancer-related mortality worldwide, accounting for 5.6% of all cancer-attributable deaths [1,2]. Esophagectomy remains the cornerstone of multidisciplinary treatment for resectable disease; however, it is also one of the most complex procedures in gastrointestinal surgery, with reported 30- and 90-day mortality rates of 2.4% and 4.5%, respectively [3].

Despite advances in surgical techniques, perioperative optimization, and postoperative management, morbidity after curative esophagectomy remains high, approaching 50% even in high-volume centers [1,4,5]. More than half of patients experience multiple postoperative complications, and 20–30% of these are classified as severe [3,4,6,7,8]. Postoperative complications are associated with reduced long-term survival, prolonged hospital stay, impaired quality of life, and increased healthcare costs [9].

Several patient-related and disease-related factors have been associated with increased risk of postoperative complications and reduced survival after esophagectomy. These include advanced age, low body mass index (BMI), impaired nutritional status reflected by hypoalbuminemia, systemic inflammation, and advanced tumor stage, particularly lymph node involvement. These factors may influence both perioperative resilience and long-term oncological outcomes, highlighting the importance of comprehensive preoperative risk stratification.

Infectious complications are among the most clinically relevant postoperative events due to their frequency and severity. Approximately 25% of patients develop infectious complications, most commonly pneumonia or sepsis secondary to anastomotic leakage [10]. These events have been linked to decreased survival and a higher incidence of metastatic disease compared with patients without postoperative infections [11]. Sepsis and septic shock, in particular, are associated with markedly worse outcomes in critically ill patients, with mortality rates up to twofold higher and in-hospital mortality reaching 46% [12,13].

Reducing postoperative morbidity and mortality requires a multidisciplinary approach encompassing careful patient selection, optimization of preoperative status, meticulous surgical technique, and intensive postoperative care. In this context, the aim of the present study was to identify perioperative factors associated with the development of septic shock following esophagectomy for esophageal cancer and, secondarily, to evaluate its impact on short- and long-term survival. From a personalized medicine perspective, identifying patient-specific perioperative risk factors may improve risk stratification and enable more individualized management strategies. Integrating clinical, nutritional, and oncological variables may help better characterize individual patient risk and guide tailored perioperative approaches aimed at reducing severe complications after esophagectomy.

## 2. Materials and Methods

### 2.1. Study Design and Population

We conducted a retrospective observational study including all consecutive adult patients who underwent esophagectomy with curative intent for esophageal carcinoma at the Department of Surgery of Hospital General Universitario Gregorio Marañón between January 2015 and December 2024. This study was approved by the institutional Research Ethics Committee (3 June 2024; approval number 11/2024), which waived the requirement for written informed consent due to the retrospective design and exclusive use of previously recorded clinicopathological data. All procedures were conducted in accordance with the Declaration of Helsinki.

**Figure 1:**
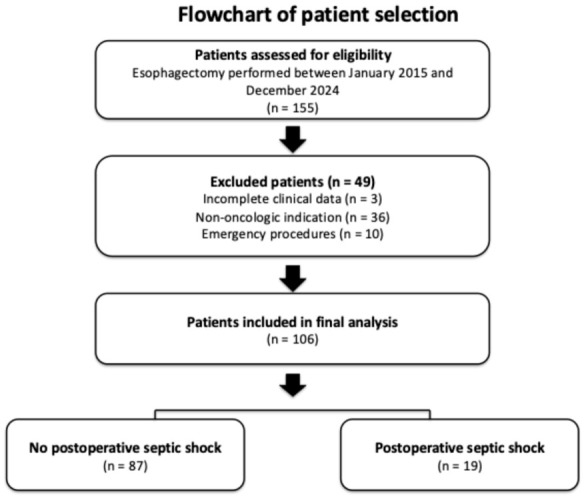
Flowchart of patient inclusion and exclusion.

Eligible patients were aged ≥18 years, had histopathological confirmation of esophageal malignancy, and underwent surgical treatment with curative intent. Exclusion criteria included: (1) incomplete clinical data, (2) non-oncologic indications for esophagectomy, and (3) emergency procedures. A flowchart summarizing patient inclusion and exclusion is provided in the Figure 1.

Patients with missing or incomplete clinical data insufficient for analysis were excluded. Emergency esophageal procedures and cases in which malignancy could not be confirmed as the indication for esophagectomy were also excluded.

### 2.2. Data Collection and Variables

Clinical data were extracted from electronic medical records. Demographic variables included age, sex, body mass index (BMI), American Society of Anesthesiologists (ASA) physical status classification, smoking status, alcohol consumption, and relevant comorbidities (arterial hypertension, diabetes mellitus, chronic pulmonary disease, chronic kidney disease, and ischemic heart disease).

Oncological variables included tumor location (upper, middle, or lower esophagus; esophageal or gastroesophageal junction), histological subtype, clinical stage, and receipt of neoadjuvant or adjuvant therapy. Pathological staging was performed according to the eighth edition of the American Joint Committee on Cancer (AJCC) TNM classification system [14].

Surgical procedures were classified as Ivor Lewis (intrathoracic anastomosis) or McKeown (cervical anastomosis). Abdominal access was achieved via laparoscopy or laparotomy, and thoracic access via video-assisted thoracoscopy or thoracotomy. Procedures were categorized as open, minimally invasive, or hybrid based on the combination of surgical approaches used.

Intraoperative variables included duration of surgery and anesthesia, use of one-lung ventilation, type of analgesia (epidural or intravenous), intraoperative fluid administration, blood transfusions, and use of vasoactive agents.

Laboratory parameters were collected preoperatively and at admission to the postoperative intensive care unit (PICU), including hemoglobin, leukocyte, lymphocyte, and platelet counts, as well as serum creatinine, glucose, albumin, total protein, C-reactive protein, procalcitonin, and lactate levels.

### 2.3. Outcomes and Definitions

The primary outcome was the development of postoperative septic shock, defined according to the Sepsis-3 criteria [15] as sepsis requiring vasopressor therapy to maintain a mean arterial pressure ≥ 65 mmHg and a serum lactate level > 2 mmol/L despite adequate fluid resuscitation.

Postoperative septic shock was defined according to Sepsis-3 criteria as sepsis requiring vasopressor therapy to maintain mean arterial pressure ≥ 65 mmHg and serum lactate > 2 mmol/L despite adequate fluid resuscitation. Management of sepsis and septic shock followed the Surviving Sepsis Campaign 2021 international guidelines, including early antibiotic therapy, source control, and hemodynamic support.

In the postoperative intensive care unit (PICU), sepsis and septic shock were managed according to a standardized institutional protocol based on international guidelines [16], including early resuscitation, source control, empiric antibiotic therapy adjusted to microbiological findings, and advanced hemodynamic and respiratory support.

Postoperative complications were prospectively recorded and classified according to the Esophagectomy Complications Consensus Group (ECCG) definitions (3). Complications were grouped into pulmonary, cardiovascular, gastrointestinal, infectious, neurological, and surgery-specific categories, including anastomotic leakage, tracheoesophageal fistula, chylothorax, pneumothorax, hemorrhagic shock, and surgical site infection.

Secondary outcomes included length of stay in the PICU and hospital, readmission to the PICU, and in-hospital mortality. Long-term follow-up was performed to determine survival status at the time of study closure.

### 2.4. Statistical Analysis

Statistical analyses were performed using SPSS software (version 25; IBM Corp., Armonk, NY, USA). Categorical variables are presented as absolute frequencies and percentages and were compared using the chi-square test or Fisher’s exact test, as appropriate. Continuous variables are reported as median and interquartile range (IQR) due to non-normal distributions and were compared between groups using the Mann–Whitney U test.

A two-sided *p* value < 0.05 was considered statistically significant. Analyses primarily focused on identifying differences between patients who developed postoperative septic shock and those who did not, using an exploratory approach to evaluate clinical, perioperative, laboratory, and oncological variables.

To identify factors independently associated with postoperative septic shock, a binary logistic regression analysis was performed. Variables showing an association with septic shock in univariate analyses (*p* < 0.10), as well as variables considered clinically relevant, were considered for inclusion. Given the limited number of septic shock events, the number of variables included in the multivariable model was deliberately restricted to minimize the risk of overfitting. A Supplementary Table (Table S1) provides a detailed overview of all variables considered for multivariable analysis and the reasons for their inclusion or exclusion.

The final model included two variables—serum albumin level at PICU admission and pathological lymph node involvement (N stage)—which demonstrated the strongest and most consistent associations with the outcome.

### 2.5. Follow-Up

Follow-up was performed through review of electronic medical records and national/regional survival registries. The maximum observed follow-up was 3116 days (approximately 103 months), allowing for long-term outcome assessment.

## 3. Results

### 3.1. Baseline Characteristics and Comorbidities

A total of 106 patients who underwent esophagectomy with curative intent were included in the analysis; 19 patients (17.9%) developed postoperative septic shock. Baseline demographic characteristics and comorbidities were largely comparable between patients who did and did not develop septic shock. However, patients in the septic shock group had significantly lower body weight and body mass index (BMI) compared with those without septic shock (Table 1).

### 3.2. Laboratory Parameters

Preoperative and early postoperative laboratory parameters are summarized in Table 2. Before surgery, patients who developed postoperative septic shock had significantly lower serum albumin levels and a higher neutrophil-to-lymphocyte ratio compared with those who did not develop septic shock. At admission to the postoperative intensive care unit (PICU), lower albumin levels persisted in the septic shock group. In addition, these patients exhibited higher procalcitonin levels and a greater perioperative deterioration in renal function at PICU admission relative to preoperative values (Table 2).

### 3.3. Oncological Characteristics

Overall, oncological and tumor-related characteristics were comparable between patients who developed postoperative septic shock and those who did not. However, lymph node involvement was significantly more advanced in the septic shock group (*p* < 0.001) (Table 3).

### 3.4. Intraoperative Factors

Most procedures were performed using a minimally invasive or hybrid approach. No significant differences were observed between patients who developed postoperative septic shock and those who did not with respect to any of the intraoperative surgical or anesthetic variables analyzed (Table 4).

### 3.5. Postoperative Course

Patients who developed postoperative septic shock experienced significantly higher rates of major postoperative complications. These included ventilator-associated pneumonia (OR 10.0; 95% CI 2.1–46.6), acute respiratory distress syndrome (OR 9.58; 95% CI 2.4–38.6), pleural effusion (OR 10.4; 95% CI 3.1–34.7), new-onset atrial fibrillation (OR 9.8; 95% CI 2.9–33.6), acute kidney failure (OR 6.3; 95% CI 2.0–19.8), delirium (OR 4.0; 95% CI 1.2–13.1), and readmission to the postoperative intensive care unit (PICU) (OR 2.99; 95% CI 1.0–8.6).

Surgical complications were also significantly more frequent in the septic shock group, particularly postoperative hemorrhage (OR 11.33; 95% CI 1.9–67.5) and anastomotic leakage (OR 9.3; 95% CI 2.8–30.8). Anastomotic leakage was the main identifiable source of infection in patients who developed septic shock, suggesting its role as a primary triggering factor. Detailed postoperative medical and surgical complications are summarized in Table 5.

### 3.6. Causes of Septic Shock

The main precipitating factor for septic shock was anastomotic leakage, which acted as a primary source of infection. Other contributing causes included pulmonary infections (e.g., pneumonia), bacteremia, and intra-abdominal infections when identifiable.

### 3.7. Management of Complications

Management of anastomotic leakage included a combination of conservative treatment, endoscopic interventions, and surgical reintervention depending on severity. A total of 15 reinterventions were performed. Septic patients were managed according to standardized ICU protocols.

### 3.8. Multivariate Analysis and Survival

In multivariate logistic regression analysis, two variables were independently associated with the development of postoperative septic shock: lower serum albumin levels at admission to the postoperative intensive care unit (PICU) and advanced lymph node involvement. Lower albumin levels at PICU admission were associated with an increased risk of septic shock (OR 0.54; 95% CI 0.29–0.99; *p* = 0.003), as was advanced nodal stage (N2/N3) (OR 4.98; 95% CI 1.36–18.3; *p* = 0.016).

**Figure 2:**
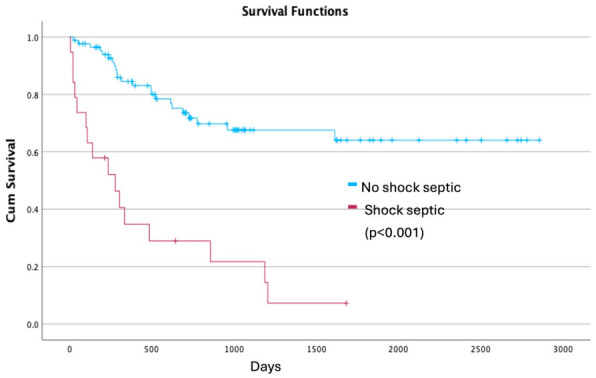
Kaplan–Meier overall survival according to postoperative septic shock in the entire cohort.

**Figure 3:**
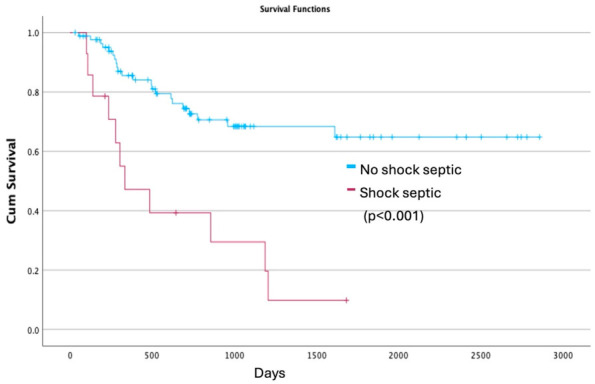
Kaplan–Meier overall survival among patients discharged alive, according to postoperative septic shock.

Survival analyses demonstrated a marked impact of postoperative septic shock on long-term outcomes. In the analysis including all patients undergoing esophagectomy, mean survival was 476 days (SE 124) in patients who developed septic shock compared with 2211 days (SE 160) in those who did not (log-rank *p* < 0.001). In a second analysis restricted to patients discharged alive after surgery, mean survival remained significantly shorter in the septic shock group (637 days [SE 147] vs. 2236 days [SE 160]; log-rank *p* < 0.001) (Figure 2 and Figure 3).

## 4. Discussion

Septic shock represents the most severe form of infection-related organ dysfunction and, in the postoperative setting, is associated with substantial mortality. The present study confirms that postoperative septic shock following esophagectomy is a complication with major clinical impact, leading to both high in-hospital mortality and markedly reduced long-term survival. Although no universally accepted system exists to comprehensively grade the occurrence and severity of all esophagectomy-related complications [3], septic shock is a clearly defined entity that is typically managed in specialized intensive care units according to well-established guidelines, thereby facilitating meaningful comparisons across centers. Our findings are consistent with previous studies showing that severe infectious complications—particularly anastomotic leakage—adversely affect both short-term clinical outcomes and long-term oncological prognosis [11,17].

Anastomotic leakage remains one of the most serious surgical complications after esophagectomy, with reported incidence rates ranging from 0% to 30% [18]. Clinical presentation varies widely, from subclinical findings to fulminant sepsis [19]. While anastomotic leakage has been extensively studied, data specifically focusing on patients who develop particularly severe clinical courses are limited. In the present cohort, the incidence of anastomotic leakage was slightly higher than that reported in many series (approximately 30%), which may be explained by the fact that our institution is not a high-volume center for this procedure, performing fewer than 50 esophagectomies per year. In our study, anastomotic leakage was the main precipitating factor for postoperative septic shock and early postoperative mortality, reinforcing its role as a key trigger of multiorgan dysfunction, as previously reported [19,20]. Notably, anastomotic leakage should be interpreted as a precipitating cause of septic shock rather than a consequence, reinforcing its central role in postoperative deterioration. This distinction is clinically relevant, as it emphasizes the importance of early detection and management of anastomotic leakage to prevent progression to septic shock. Importantly, the clinical impact of anastomotic leakage is strongly influenced by the timing of diagnosis and initiation of treatment [21]. In addition to technical factors, patient-related variables such as malnutrition, systemic inflammation, and exposure to neoadjuvant therapy are known to impair tissue healing by affecting the integrity of the submucosal collagen matrix, thereby increasing the risk of anastomotic failure [22,23].

Patients undergoing esophagectomy frequently exhibit impaired nutritional status due to tumor-related symptoms—including dysphagia, vomiting, reduced oral intake, weight loss, and sarcopenia—as well as the adverse effects of neoadjuvant treatment [24]. In our study, markers of poor nutritional status, including lower body mass index and reduced serum albumin levels (both preoperatively and at PICU admission), were associated with the development of postoperative septic shock. However, albumin should be interpreted with caution, as levels at PICU admission may reflect not only baseline nutritional status but also early postoperative physiological deterioration, including systemic inflammation, capillary leakage, and fluid shifts. Thus, albumin likely represents a composite marker of physiological reserve rather than a purely nutritional parameter. Nevertheless, as a readily available biomarker, hypoalbuminemia may serve as a practical tool for individualized risk stratification. From a personalized medicine perspective, early identification of patients with limited physiological reserve may enable tailored perioperative strategies, including intensified nutritional optimization, closer postoperative monitoring, and earlier intervention in case of clinical deterioration.

Importantly, preoperative albumin may be particularly useful because it is available before surgery, when risk remains modifiable. In this context, hypoalbuminemia could help identify patients who may benefit from structured prehabilitation programs, including early dietitian referral, individualized nutritional supplementation, physical exercise, and optimization during neoadjuvant treatment. Therefore, beyond risk prediction, preoperative albumin may serve as a practical trigger for targeted interventions aimed at improving physiological reserve before esophagectomy.

We also observed that early postoperative deterioration in renal function was associated with the development of septic shock. Acute kidney injury is a frequent complication after esophagectomy [25] and is primarily related to perioperative hypoperfusion, systemic inflammation, and the neuroendocrine stress response. Importantly, acute kidney injury has been proposed as a sentinel complication that may precipitate dysfunction in distant organs, thereby increasing postoperative morbidity and prolonging hospital stay [26]. In this context, perioperative monitoring of renal function—particularly serum creatinine—should be considered a fundamental component of postoperative surveillance. Our findings suggest that patients who developed septic shock may have experienced greater surgical stress, characterized by hypoperfusion, inflammatory activation, and neuroendocrine dysregulation, which adversely influenced their postoperative course. Such vulnerability may be detected early through simple laboratory assessment at admission to the postoperative intensive care unit (PICU).

Postoperative hemorrhage after esophagectomy is an uncommon but potentially life-threatening complication. The incidence observed in our cohort was comparable to that reported in previous studies [27] and has been linked to the presence of anastomotic leakage [22]. Notably, in our series, all cases of hemorrhagic shock occurred in patients with anastomotic dehiscence, further supporting the close interplay between major surgical complications and the development of severe postoperative deterioration.

Lymphadenectomy is a critical component of curative esophageal cancer surgery and has been associated with improved oncological outcomes; however, it is technically demanding and carries a substantial risk of severe postoperative complications [28]. Advanced lymph node involvement reflects a higher tumor burden and often necessitates more extensive resections, aggressive lymphadenectomy, and increased tissue manipulation, which may predispose patients to severe infectious complications. Previous studies have shown that advanced nodal disease (N2/N3) is independently associated with higher rates of recurrence and severe postoperative morbidity [29]. This association may also be partially explained by the increased physiological frailty and reduced physiological reserve of patients with more advanced oncological disease. These findings also support the concept of a more personalized perioperative risk assessment in esophageal cancer surgery. Integrating oncological factors such as lymph node involvement with clinical and laboratory variables may help identify patients at particularly high risk of severe postoperative complications, thereby facilitating individualized perioperative decision-making and surveillance strategies. These findings further support the role of advanced nodal disease as a marker of increased surgical complexity, systemic inflammation, and reduced physiological reserve.

Beyond its immediate clinical impact—driven by multiorgan failure, need for reintubation, vasoactive support, and frequent surgical or endoscopic reinterventions—postoperative septic shock appears to have lasting consequences. This likely explains the markedly increased in-hospital mortality observed in our cohort. More importantly, our data indicate that even among patients discharged alive, septic shock was associated with significantly reduced long-term survival. Survivors of severe surgical sepsis are known to experience persistent physical, cognitive, and psychosocial impairment, with high rates of functional dependence, post-intensive care syndrome, and post-traumatic stress symptoms extending well beyond hospital discharge [30,31]. Consistent with these observations, a recent meta-analysis reported that severe postoperative complications are associated with an average reduction of 8.6 months in 5-year overall survival [1], underscoring their role not only as acute adverse events but also as modifiers of long-term oncological prognosis. Nevertheless, it should be acknowledged that other studies have failed to demonstrate a significant association between postoperative complications and long-term survival [17,32,33], highlighting the ongoing debate in this field.

From a personalized medicine perspective, our findings highlight the potential of integrating simple perioperative variables into individualized risk prediction models. Readily available markers such as serum albumin and pathological nodal stage may serve as practical tools to identify patients at high risk of severe postoperative complications, including septic shock. This approach could enable risk-adapted perioperative pathways, including intensified nutritional optimization, closer postoperative monitoring, and early therapeutic interventions. Ultimately, incorporating such predictors into clinical decision-making may contribute to more precise, patient-centered care in esophageal cancer surgery.

This study has several limitations. Its retrospective, single-center design limits generalizability and causal inference, and the relatively small number of septic shock cases restricts statistical power. Additionally, heterogeneity in surgical expertise over the long study period may have influenced outcomes. Some clinically relevant variables could not be included due to missing data or risk of overfitting. Furthermore, adjusted survival analyses were not feasible. These findings should therefore be interpreted with caution and confirmed in larger, multicenter studies. In addition, surgical practice evolved during the study period (2015–2024), with progressive adoption of minimally invasive and hybrid techniques, refinement of perioperative care pathways, and increasing multidisciplinary experience. Although these temporal changes reflect real-world practice, they may also have introduced a learning-curve effect that could have influenced postoperative outcomes. This should be considered when interpreting the results.

In conclusion, postoperative septic shock after esophagectomy is a severe complication associated with markedly increased in-hospital mortality and reduced long-term survival. Hypoalbuminemia at ICU admission and advanced lymph node involvement were independently associated with its development. These findings support the implementation of personalized perioperative risk stratification strategies, integrating clinical, nutritional, and oncological variables to identify high-risk patients. Such an approach may enable tailored perioperative management, including targeted nutritional optimization and enhanced postoperative surveillance, ultimately contributing to improved outcomes in esophageal cancer surgery.

## Figures and Tables

**Table 1:** Baseline demographic and clinical characteristics according to postoperative septic shock status.

Variable	Total (*n* = 106)	No Septic Shock (*n* = 87)	Septic Shock (*n* = 19)	*p* Value
**Demographics**				
Age, years	59.5 (52–68)	60 (52–68)	58.0 (52–68)	0.789
Weight, kg	72.4 (62–83)	74 (63–85)	65 (59–73)	0.046
Height, cm	170 (166–175)	170 (167–175)	168 (160–170)	0.096
BMI, kg/m^2^	24.9 (22–28)	25.5 (22–28)	23.4 (20–26)	0.036
Male sex, *n* (%)	92 (86.8)	77 (88.5)	15 (78.9)	0.789
**Lifestyle**				
Active alcohol use, *n* (%)	15 (14.1)	11 (12.8)	4 (21.1)	0.272
Former alcohol use, *n* (%)	11 (10.3)	10 (11.6)	1 (5.3)	0.368
Active smoker, *n* (%)	33 (31.1)	26 (30.2)	7 (36.8)	0.574
Former smoker, *n* (%)	37 (34.9)	31 (36)	6 (31.6)	0.712
Preoperative corticosteroid therapy, *n* (%)	8 (7.5)	7 (8.1)	1 (5.3)	0.556
**Comorbidities**				
ASA physical status, *n* (%)				0.078
• I	1 (0.9)	1 (1.2)	0 (0)	
• II	40 (37.7)	28 (32.6)	12 (63.2)	
• III	61 (57.5)	54 (62.8)	6 (31.6)	
• IV	4 (3.7)	3 (3.5)	1 (5.3)	
COPD, *n* (%)	4 (3.7)	3 (3.5)	1 (5.3)	0.556
OSA, *n* (%)	2 (1.8)	2 (2.3)	0 (0)	0.502
DMNID, *n* (%)	13 (12.2)	13 (15.1)	0 (0)	0.062
DMID, *n* (%)	3 (2.8)	2 (2.3)	1 (5.3)	0.454
CKD, *n* (%)	6 (5.6)	5 (5.8)	1 (5.3)	0.703
Hypertension, *n* (%)	44 (41.5)	37 (43.0)	7 (36.8)	0.621
Dyslipidemia, *n* (%)	41 (38.6)	32 (37.2)	9 (47.4)	0.411
Ischemic heart disease, *n* (%)	6 (5.6)	6 (7.0)	0 (0)	0.292
CHF, *n* (%)	1 (0.9)	1 (1.2)	0 (0)	0.819
Atrial fibrillation, *n* (%)	5 (4.7)	5 (5.8)	0 (0)	0.361
Hypothyroidism, *n* (%)	1 (0.9)	1 (1.2)	0 (0)	0.819
Cerebrovascular disease, *n* (%)	3 (2.8)	3 (3.5)	0 (0)	0.546
Chronic liver disease, *n* (%)	4 (3.7)	3 (3.5)	1 (5.3)	0.556
PVD, *n* (%)	8 (7.5)	7 (8.1)	1 (5.3)	0.556

Data are presented as median (interquartile range) or number (%). BMI: body mass index; ASA: American Society of Anesthesiologists; COPD: chronic obstructive pulmonary disease; OSA: obstructive sleep apnea; DMID: diabetes mellitus, insulin-dependent; DMNID: diabetes mellitus, non-insulin-dependent; CKD: chronic kidney disease; CHF: congestive heart failure; and PVD: peripheral vascular disease.

**Table 2:** Preoperative and early postoperative laboratory parameters according to postoperative septic shock status.

Variable	Total (*n* = 106)	No Septic Shock (*n* = 87)	Septic Shock (*n* = 19)	*p* Value
Preoperative				
Hemoglobin, g/dL	12.6 (11.8–13.7)	12.6 (11.8–13.6)	12.6 (11.0–14.1)	0.745
Leukocytes, ×10^9^/L	6.2 (4.7–7.9)	6 (4.7–7.9)	7 (4.2–10.1)	0.446
Lymphocytes, ×10^9^/L	1.2 (0.9–1.8)	1.3 (0.9–1.8)	1.0 (0.7–1.4)	0.155
Platelets, ×10^9^/L	210 (149–254)	212 (160–254)	209 (140–254)	0.705
INR	1.02 (0.97–1.09)	1.02 (0.97–1.1)	1 (0.94–1.07)	0.617
Fibrinogen, mg/dL	496 (437–583)	495 (438–578)	505 (432–760)	0.509
Glucose, mg/dL	97.0 (89.0–113)	97 (89–113)	99 (89–121)	0.863
Creatinine, mg/dL	0.77 (0.65–0.89)	0.79 (0.65–0.89)	0.73 (0.62–0.92)	0.714
Albumin, g/dL	4.1 (3.7–4.3)	4.1 (3.8–4.3)	3.85 (3.5–4.1)	0.039
Total proteins, g/dL	6.6 (6.3–7)	6.6 (6.3–7)	6.7 (6.2–7.2)	0.551
C-reactive protein, mg/L	1.5 (0.2–6.7)	0.8 (0.2–6.2)	10.7 (0.85–27)	0.496
Procalcitonin, ng/mL	0.07 (0.03–0.11)	0.05 (0.03–0.10)	0.11 (0.07–0.12)	0.179
Neutrophil-to-lymphocyte ratio	4.9 (3.5–8)	4.7 (3.3–6.2)	6.8 (4.1–10.1)	0.049
Platelet-to-lymphocyte ratio	167 (104–263)	159 (104–236)	238 (110–310)	0.227
Albumin-to-lymphocyte ratio	3.3 (2.2–4.5)	3.2 (2.1–4.4)	3.6 (2.2–5.1)	0.398
PICU admission				
Hemoglobin, g/dL	11.6 (10.5–12.6)	11.5 (10.6–12.4)	11.8 (9.6–13.1)	0.780
Platelets, ×10^9^/L	182 (147–224)	185 (152–223)	166 (119–248)	0.632
INR	1.11 (1.03–1.21)	1.11 (1.03–1.21)	1.10 (0.97–1.23)	0.492
Fibrinogen, mg/dL	497 (431–609)	511 (446–597)	438 (357–693)	0.326
Lymphocytes, ×10^9^/L	0.6 (0.4–1.0)	0.7 (0.4–1.1)	0.5 (0.3–0.8)	0.094
Leukocytes, ×10^9^/L	10.2 (7.9–12.5)	10.1 (7.9–12.7)	10.6 (8.6–12)	0.822
Glucose, mg/dL	144 (119–165)	144 (115–166)	147 (124–165)	0.334
Creatinine, mg/dL	0.76 (0.6–0.9)	0.75 (0.6–0.9)	0.8 (0.7–0.9)	0.206
Albumin, g/dL	3.2 (3–3.5)	3.3 (3.1–3.5)	3.1 (2.3–3.4)	0.024
Total proteins, g/dL	5.5 (5–5.7)	5.5 (5.1–5.7)	5.4 (4.7–6)	0.914
C-reactive protein, mg/L	21.7 (4.4–61)	21.7 (5.7–60)	57.6 (0.6–115)	0.956
Procalcitonin, ng/mL	0.1 (0.04–0.17)	0.07 (0.03–0.14)	0.19 (0.15–2.17)	0.044
Lactate, mmol/L	2.05 (1.3–3.6)	1.9 (1.3–3.5)	2.8 (1.8–3.7)	0.306
Neutrophil-to-lymphocyte ratio	15.3 (10–26)	14.7 (9–24)	17.5 (14–28)	0.064
Albumin-to-lactate ratio	1.58 (0.9–2.4)	1.68 (0.9–2.5)	1.03 (0.8–1.8)	0.064
Platelet-to-lymphocyte ratio	274.29 (175.45–475.56)	252.43 (174–446)	380 (181–595)	0.145
Albumin-to-lymphocyte ratio	5.33 (2.9–7.4)	4.93 (2.9–7.2)	5.75 (3–10.5)	0.446
Perioperative changes				
Albumin-to-lymphocyte ratio (preop/PICU)	0.8 (0.5–1.1)	0.8 (0.5–1.1)	0.7 (0.5–1.44)	0.729
Creatinine change, mg/dL	0.04 (−0.06–0.13)	0.05 (−0.04–0.14)	0.06 (−0.19–0.13)	0.041

Data are presented as median (interquartile range). PICU: postoperative intensive care unit; INR: international normalized ratio.

**Table 3:** Oncologic and tumor characteristics according to postoperative septic shock status.

Variable	Total (*n* = 106)	No Septic Shock (*n* = 87)	Septic Shock (*n* = 19)	*p* Value
Neoadjuvant chemotherapy *n* (%)	68 (64.1)	55 (83.3)	13 (81.3)	0.547
Neoadjuvant radiotherapy *n* (%)	40 (37.7)	30 (34.9)	10 (52.6)	0.149
Pathologic T stage (T), *n* (%)				0.791
• T1	9 (8.5)	8 (9.2)	1 (5.3)	
• T2	35 (33)	27 (31)	8 (42.1)	
• T3	48 (45.3)	40 (46)	8 (42.1)	
• T4	14 (13.2)	12 (13.8)	2 (10.5)	
Pathologic N stage (N), *n* (%)				0.009
• N0	14 (13.2)	13 (14.9)	1 (5.3)	
• N1	31 (29.2)	29 (33.3)	2 (10.5)	
• N2	42 (39.6)	34 (39.1)	8 (42.1)	
• N3	19 (17.9)	11 (12.6)	8 (42.1)	
Pathologic M stage (M), *n* (%)				0.367
• M0	94 (88.7)	78 (89.7)	16 (84.2)	
• M1	12 (11.3)	9 (10.3)	3 (15.8)	
Tumor location, *n* (%)				0.763
• Distal	79 (74.5)	65 (74.7)	14 (73.7)	
• Mid–distal	14 (13.2)	12 (13.8)	2 (10.5)	
• Middle	7 (6.6)	6 (6.9)	1 (5.3)	
• Proximal	6 (5.7)	4 (4.6)	2 (10.5)	

Data are presented as *n* (%). TNM staging according to the AJCC classification.

**Table 4:** Intraoperative surgical and anesthetic variables according to postoperative septic shock status.

Variable	Total (*n* = 106)	No Septic Shock (*n* = 87)	Septic Shock (*n* = 19)	*p* Value
Surgical approach, *n* (%)				0.594
• Open	17 (16)	12 (14)	5 (26.3)	
• Hybrid	44 (41.5)	35 (40.7)	9 (47.4)	
• Minimally invasive	45 (42.5)	40 (46.5)	5 (26.3)	
Feeding jejunostomy, *n* (%)	86 (81.1)	70 (81.4)	16 (88.9)	0.354
Intraoperative radiotherapy, *n* (%)	13 (12.3)	10 (11.9)	3 (15.8)	0.430
Surgical procedure, *n* (%)				0.37
• Ivor Lewis	60 (56.6)	51(58.6)	9 (47.4)	
• McKeown	46 (43.4)	36 (41.4)	10 (52.6)	
Operative times				
Duration of surgery, min	450 (370–525)	454 (384–528.5)	435 (320–470)	0.192
Duration of anesthesia, min	579 (509–663)	594 (510–667)	567 (450–663)	0.317
Airway/ventilation				
One-lung ventilation	81 (76.4)	69 (79.3)	12 (63.2)	0.133
Bronchial blocker used, *n* (%)	59 (55.7)	48 (55.2)	11 (57.9)	0.956
Hemodynamic/anesthesia				
Epidural analgesia, *n* (%)	74 (69.8)	61 (70.1)	13 (68.4)	0.828
Use of vasoactive drugs, *n* (%)	35 (33)	26 (29.9)	9 (47.4)	0.142
Extubated in operating room, *n* (%)	89 (84)	74 (85.1)	15 (78.9)	0.36
Red blood cell transfusion, *n* (%)	2 (1.9)	1 (1.2)	1 (5.3)	0.342
Crystalloids, mL	2200 (1500–2500)	2100 (1500–2500)	2500 (2000–2500)	0.858
Colloids, mL	250 (0–500)	150 (0–500)	500 (500–500)	0.1

Data are presented as *n* (%) or median (interquartile range). OLV: one-lung ventilation.

**Table 5:** Postoperative complications and outcomes according to postoperative septic shock status.

Variable	Total (*n* = 106)	No Septic Shock (*n* = 87)	Septic Shock (*n* = 19)	*p* Value
Pulmonary complications				
Atelectasis *n* (%)	12 (11.3)	9 (10.3)	3 (15.8)	0.367
Pleural effusion *n* (%)	38 (35.8)	23 (26.4)	15 (78.9)	<0.001
Hospital-acquired pneumonia *n* (%)	35 (33.0)	26 (29.9)	9 (47.4)	0.142
Ventilator-associated pneumonia *n* (%)	8 (7.5)	3 (3.4)	5 (26.3)	0.004
Empyema *n* (%)	8 (7.5)	5 (5.7)	3 (15.8)	0.152
Pulmonary embolism *n* (%)	2 (1.9)	2 (2.3)	0 (0)	0.672
Acute respiratory distress syndrome *n* (%)	10 (9.4)	4 (4.6)	6 (31.6)	<0.001
Cardiovascular/Neurologic				
Acute myocardial infarction *n* (%)	1 (0.9)	1 (1.1)	0 (0)	0.821
New-onset atrial fibrillation *n* (%)	14 (13.2)	6 (6.9)	8 (42.1)	<0.001
Cardiac arrest *n* (%)	2 (1.9)	0 (0)	2 (10.5)	0.031
Stroke *n* (%)	1 (0.9)	1 (1.1)	0 (0)	0.821
Delirium *n* (%)	15 (14.2)	9 (10.3)	6 (31.6)	0.016
Renal/Infectious				
Acute kidney injury *n* (%)	17 (16.0)	9 (10.3)	8 (42.1)	<0.001
Bacteremia *n* (%)	8 (7.5)	5 (5.7)	3 (15.8)	0.152
Sepsis (non-shock) *n* (%)	33 (31.1)	26 (29.9)	7 (36.8)	0.553
Surgical complications				
Ileus *n* (%)	11 (10.4)	7 (8.0)	4 (21.1)	0.107
Biliary leak *n* (%)	2 (1.9)	1 (1.1)	1 (5.3)	0.328
Anastomotic leak *n* (%)	40 (37.7)	25 (28.7)	15 (78.9)	<0.001
Bronchoesophageal fistula *n* (%)	7 (6.6)	5 (5.7)	2 (10.5)	0.367
Chylothorax *n* (%)	3 (2.8)	1 (1.1)	2 (10.5)	0.082
Pneumothorax *n* (%)	8 (7.5)	6 (6.9)	2 (10.5)	0.438
Surgical site infection *n* (%)	9 (8.5)	7 (8.0)	2 (10.5)	0.506
Hemorrhagic shock *n* (%)	6 (5.7)	2 (2.3)	4 (21.1)	0.004
Resource use/Outcomes				
PICU length of stay (days)	5 (2–10.5)	4 (2–7.3)	8 (4–26)	0.008
Hospital length of stay (days)	21 (13–36)	19 (13–31.5)	37 (21–72)	0.011
PICU readmission *n* (%)	25 (23.6)	17 (19.5)	8 (42.1)	0.036
In-hospital mortality *n* (%)	7 (6.6)	1 (1.1)	6 (31.6)	<0.001

Data are presented as median (IQR) or *n* (%). ARDS, acute respiratory distress syndrome; PICU, postoperative intensive care unit.

## Data Availability

The original contributions presented in this study are included in the article. Further inquiries can be directed to the corresponding author.

## Supplementary Materials

The following supporting information can be downloaded at: https://www.mdpi.com/article/10.3390/jpm16050251/s1, Table S1: Variables considered for multivariable analysis.
