# Supplementary material for: Postoperative Septic Shock After Esophagectomy for Esophageal Cancer: Risk Factors and Impact on Short- and Long-Term Survival
Source: J Pers Med. 2026 May 4;16(5):251. doi: 10.3390/jpm16050251 (PMC13208200; doi:10.3390/jpm16050251)
Supplement: Supplementary file 1 [file jpm-16-00251-s001.zip › jpm-4229335-supplementary.pdf]

Supplementary Table S1. Variables considered for multivariable logistic regression analysis and reasons for exclusion

Variable	Univariate p-value	Clinical relevance	Included in multivariable model	Reason for exclusion
BMI	0.036	Yes	No	Limited events / avoid overfitting
Preoperative albumin	0.039	Yes	No	Collinearity with PICU albumin
PICU albumin	0.024	Yes	Yes	Strongest association
Nodal stage	0.009	Yes	Yes	Strong clinical relevance
Neutrophil-to-lymphocyte ratio	0.049	Yes	No	Limited events
Procalcitonin	0.044	Yes	No	Redundancy with infection severity
Creatinine change	0.041	Yes	No	Limited events
Anastomotic leakage	<0.001	Yes	No	Considered downstream event
Operative time	NS	Moderate	No	Not significant
Age	NS	Yes	No	Not significant

Note: Due to the limited number of septic shock events (n = 19), the number of variables included in the multivariable model was restricted to avoid overfitting. Variables were selected based on both statistical significance and clinical relevance.
